# Incremental Healthcare Expenditures Associated with Thyroid Disorders among Individuals with Diabetes

**DOI:** 10.1155/2012/418345

**Published:** 2012-12-11

**Authors:** Amit D. Raval, Usha Sambamoorthi

**Affiliations:** Department of Pharmaceutical Systems and Policy, School of Pharmacy, West Virginia University, P.O. Box 9510, Morgantown, WV-26505, USA

## Abstract

*Objective*. To estimate incremental healthcare expenditures associated with thyroid disorders among individuals with diabetes. *Research Design and Methods*. Cross-sectional study design with data on adults over 20 years of age with diabetes (N = 4, 490) from two years (2007 and 2009) of the Medical Expenditure Panel Survey (MEPS) was used. Ordinary least square regressions on log-transformed total expenditures and type of healthcare expenditures (inpatient, emergency room, outpatient, prescription drug, and other) were performed to estimate the incremental expenditures associated with thyroid disorders after controlling for demographic, socioeconomic, health status, lifestyle risk factors, macrovascular comorbid conditions (MCCs), and chronic conditions (CCs). *Results*. Among individuals with diabetes, those with thyroid disorders had significantly greater average annual total healthcare expenditures ($15,182) than those without thyroid disorders ($11,093). Individuals with thyroid disorders had 34.3% greater total healthcare expenditures compared to those without thyroid disorders, after controlling for demographic, socio-economic, and perceived health status. Furthermore, controlling for CCs and MCCs, this increase in expenditures was reduced to 21.4%. *Conclusions*. Among individuals with diabetes, thyroid disorders were associated with greater healthcare expenditures; such excess expenditures may be due to CCs and MCCs. Comanagement of CCs and reducing MCCs may be a pathway to reduce high healthcare expenditures.

## 1. Introduction

In 2011, approximately 20 million Americans were affected by thyroid disorders [1]. Individuals with diabetes are at greater risk for developing thyroid disorders and have a higher prevalence of thyroid disorders compared to the general population. Prevalence of thyroid disorders among individuals with diabetes ranges from 13%–32% compared to 5%–8% in the general population [2–5]. Although hypothyroidism is a common disorder among individuals with diabetes [2–5], hyperthyroidism is also prevalent and is estimated to be around 12% [6].

As thyroid hormones affect glucose metabolism [7–10], individuals with diabetes and hypothyroidism may suffer from frequent recurrent hypoglycemic episodes [11] and insulin resistance [12, 13]. Thyroid hormones also affect body weight, placing individuals with thyroid disorders at increased risk of obesity and negative consequences related to obesity [8, 12, 13]. Individuals with diabetes and hyperthyroidism may suffer from hyperglycemia possibly due to increased metabolism of insulin, increase in glucose absorption from gut, increase in endogenous glucose production, lipolysis resulted in hepatic gluconeogenesis, increase in growth hormones, glucagon and catecholamine levels [7, 9, 14–18]. 

Elevated risk of microvascular complications such as retinopathy and nephropathy among individuals with diabetes and subclinical hypothyroidism compared to individuals with diabetes and without subclinical hypothyroidism has been documented [19–22]. In addition, thyroid disorders may lead to macrovascular complications. In a recent study, subclinical hypothyroidism was associated with increased risk of cardiovascular mortality and morbidity specifically increased incidence of atrial fibrillation [23]. In addition, thyroid disorders cooccur with many common chronic conditions, for example, depression, arthritis, and asthma [24–28] which can further complicate the management of both diabetes and thyroid disorders. 

The negative consequences of abnormally high or low levels of thyroid hormones on glycemic control, micro- and macrovascular complications can lead to greater economic burden as well. Among individuals with diabetes, macrovascular conditions are reported to be a major driver of healthcare expenditures [29–31]. However, studies that examine the excess economic burden measured by direct healthcare expenditures associated with thyroid disorders among individuals with diabetes are scarce. There is a need for studies in this area to better understand the drivers of greater healthcare expenditures among individuals with diabetes and thyroid disorders and develop strategies and programs to reduce the economic burden in this group. Therefore, the primary objective of the study is to provide cross-sectional estimates of additional healthcare expenditures associated with thyroid disorders among adults with diabetes by comparing them to those with diabetes and without thyroid disorders, using a nationally representative survey of households and families.

## 2. Methods

### 2.1. Study Design

This study utilized a cross-sectional design and compared healthcare expenditures among individuals with thyroid disorders and diabetes to individuals with diabetes without thyroid disorders. 

#### 2.1.1. Data Source

The Medical Expenditure Panel Survey (MEPS) data for the years 2007 and 2009 were used for this study. The MEPS is a set of large-scale surveys of families and individuals, their medical providers (doctors, hospitals, pharmacies, etc.), and employers across the United States of America (USA). The MEPS is cosponsored by the Agency for Healthcare Research and Quality (AHRQ) and the National Center for Health Statistics. The survey provides nationally representative estimates of healthcare use, expenditures, payment sources, health insurance coverage, health status, health conditions, and treatment for the civilian noninstitutionalized population of USA. 

For the current study, we used household and medical conditions files of the MEPS. The household component collects person-level data on demographic characteristics, health conditions, health status, use of medical services, charges and payments, access to care, employment, health insurance coverage, and income for a period of two years. Medical conditions file provides information on medical conditions that were reported by the households. The medical conditions and procedures reported by the household component respondent are usually recorded by the interviewer as verbatim text, which is then coded by professional coders to fully specifiedInternational Classification of Diseases, Ninth Revision, Clinical Modification (ICD-9-CM) codes, including medical condition and V codes [32, 33]. However, to preserve confidentiality, the MEPS data provide nearly all of the diagnosis condition codes only 3-digit ICD-9 CM codes. In addition, the MEPS data provide clinical classification codes, which are aggregated ICD-9-CM codes into clinically meaningful categories that group similar conditions. For the purpose of this study, we used both ICD-9 CM as well as clinical classification codes to identify the medical condition appropriately (see details of ICD-9CM and CCS crosswalk on: http://meps.ahrq.gov/mepsweb/data_stats/download_data/pufs/h128/h128_icd9codes.shtml).

 We included data for the years 2007 and 2009 to gain sufficient sample size. As individuals are followed for two years, to avoid including repeated observations from the same individual, we selected alternate years.

### 2.2. Study Sample

 The study sample included adults, aged 21 years or older, and reported diabetes during the calendar years and alive during the calendar years. Diabetes was identified from clinical classification codes 49 and 50 (Table 5). Detailed information on the crosswalk between ICD-9-CM codes and clinical classification codes is available elsewhere [32, 33]. The final study sample consisted of 4,490 individuals with diabetes, of whom 545 reported having thyroid disorders. 

### 2.3. Measures

#### 2.3.1. Dependent Variables

**Healthcare Expenditures: Total and Type of Expenditures**

Within MEPS, healthcare services, which were paid by third-party payers and/or individuals themselves are defined as health expenditures and reported for each year. Expenditures are also distinguished by the type of expenditures, and aggregated by inpatient care; ambulatory care, provided in offices and hospital outpatient departments; care provided in emergency departments; prescribed medications, vision, dental, home health care, and others. Sources include direct payments from individuals, private insurance, Medicare, Medicaid, Veteran Affairs, other public insurance, workers' compensation, and other sources [32, 33]. In current study, we classified the types of expenditures into: (1) inpatient; (2) emergency room; (3) outpatient; (4) prescription drugs; (5) other. Other expenditures included home health care, vision, dental, durable medical equipment, and ambulance services, orthopedic items, hearing devices, prostheses, bathroom aids, medical equipment, disposable supplies, alterations/modifications, and other miscellaneous items or services that were obtained, purchased, or rented during the year [32, 33]. Expenditures in 2007 were converted to 2009 dollars using the annual consumer price index for medical care services available from the bureau of labor statistics [34]. As expenditures data were skewed to the right, we transformed expenditures to natural logarithmic scale and we used logged expenditures as dependent variables in regression analysis.

**Key Independent Variable: Presence of Thyroid Disorders**

Thyroid disorders were identified using clinical classification code of 48, which represented all thyroid disorders such as acquired hypothyroidism (ICD-9-CM code −244), thyrotoxicosis (ICD-9-CM code −242), thyroiditis (ICD-9-CM code −245), and other disorders of thyroid (ICD-9-CM code −246) [35]. We created a dummy variable for presence of thyroid disorder for the purpose of analysis.

#### 2.3.2. Other Independent Variables

Demographic variables included gender (women, men), categories of age in years (21–49, 50–64, and 65 and +), race/ethnicity (White, African American, Latino, and other), marital status (married, widowed, separated/divorced, and never married), and area of residence (metro and nonmetro). Socioeconomic characteristics were categorized by education (less than high school, high school, and above high school) and poverty status. The poverty status variable was defined poor (less than 100% federal poverty line), near poor (100% to less than 200%), middle income (200% to less than 400%), and high income (greater than or equal to 400%) available from MEPS represented family income in relation to the federal poverty line (based on family size and composition). 

Health status was measured with widely used standard scales of perceived physical and mental health. Perceived physical and mental health status were categorized into excellent/very good, good, and fair/poor. Body mass index categories (BMI—normal or underweight, overweight, and obese), current smoking (yes/no), and vigorous physical activity at least 3 times/week (yes/no) represented lifestyle risk factors. Healthcare expenditures are often influenced by the presence of chronic physical conditions such as arthritis, chronic obstructive pulmonary disease (COPD), gastroesophageal reflux disease (GERD), other endocrine disease, and osteoporosis and mental health conditions such as depression and anxiety [31, 36–38]. Therefore, we also included the total number of cooccurring chronic conditions as one of the independent variables. For details of conditions and codes, please see Table 5.

**Macrovascular Comorbid Conditions**

To understand the association between diabetes complications and expenditures, we included macrovascular comorbid conditions as defined by Fu and colleagues [31] as one of the independent variables. The macrovascular comorbid conditions were defined using clinical classification codes and appropriate ICD-9 CM 3-digit codes, which included codes for cardiovascular disease (ischemic heart disease, congestive heart failure, aortic/visceral/peripheral aneurysms, visceral atherosclerosis, and peripheral vascular disease) and cerebrovascular disease (strokes and transient ischemic attacks).

### 2.4. Statistical Techniques

To test unadjusted differences in logged expenditures, ordinary least square regressions were used to estimate the incremental expenditures associated with thyroid disorders among individuals with diabetes. Effect estimates for continuous independent variables on the log of annual expenditures can be interpreted as percentage change for each unit of change in the independent variable. The effect of categorical variables in terms of percentage of expenditures can be estimated by exponentiating the regression coefficients of dummy variables and subtracting one (i.e., percent change = *e* ^*β*^ − 1) [39]. Therefore, in tables, we also present percent change in expenditures in addition to parameter estimates.

To understand the association between thyroid disorders expenditures among individuals with diabetes, we conducted four different regression models in which independent variables were entered in blocks. In Model 1, we controlled for age, gender and race/ethnicity. In Model 2, we adjusted for other demographic, socioeconomic, and life-style factors in addition to variables used in Model 1. In Model 3, in addition to the variables used in Model 2, we included number of cooccurring chronic conditions. In Model 4, we controlled for the presence of macrovascular comorbid conditions, in addition to all other variables included in Model 3. All analyses account for complex survey design of the MEPS and statistical testing was carried out with survey procedures in SAS 9.2. 

## 3. Results

### 3.1. Description of the Study Sample

In our study sample, 12% (*N* = 545) individuals reported having cooccurring thyroid disorders. The demographics and socio-economic characteristics, access to care, health status, and personal health practices of individuals with diabetes with or without thyroid disorders are presented in Table 1. 

Among individuals with diabetes, gender, age, race/ethnicity, marital status, poverty status, health insurance, BMI, smoking status, and physical activity were significantly different between individuals with and without thyroid disorders. For example, a significantly higher proportion of women (20%) than men (7%) reported having thyroid disorders. Similarly, a significantly higher proportion of elderly who were 65 years or older (17.8%) reported having thyroid disorders compared to young adults in the age group 21–50 years (10.1%). Compared to African Americans (8.6%), a significantly higher proportion of Whites reported having thyroid disorders (16.3%). Regarding lifestyle risk factors, a lower proportion of smokers (10.5%) reported having thyroid disorders compared to nonsmokers (15%). Individuals who performed moderate-to-vigorous physical activity at least three times a week had lower rates of thyroid disorders (12.3%) compared to individuals who did not perform moderate-to-vigorous physical activity at least three times a week (15%).

 We examined the prevalence of cooccurring chronic and macrovascular comorbid conditions (Table 2). Among individuals with diabetes, those with thyroid disorders reported significantly higher rates of cooccurring chronic conditions and macrovascular comorbid conditions compared to their counterparts without thyroid disorders. For example, 39.5% of those with thyroid disorders reported the presence of any macrovascular comorbid conditions compared to 30.6% of individuals with diabetes and without thyroid disorders. Similarly, 35.8% of those with thyroid disorders reported heart disease compared to 28% among those with diabetes and without thyroid disorders.

In terms of number chronic conditions, 14.6% of those with thyroid disorders reported 5 or more conditions compared to 6.7% among those with diabetes and without thyroid disorders (data not shown in tabular form). In addition, individuals with thyroid disorders had significantly greater number of chronic physical and mental conditions compared to individuals without thyroid disorders. 

### 3.2. Healthcare Expenditures

Among individuals with diabetes, comparisons of average total, inpatient, emergency room, outpatient, prescription drug, and other expenditures for those with and without thyroid disorders are presented in Table 3. Significant group differences in expenditures were tested using log-transformed expenditures using an OLS regression in which the presence of thyroid disorders was the independent variable. Individuals with diabetes and thyroid disorders had significantly greater total healthcare expenditures than those without thyroid disorders ($15,182 versus $11,093, *P* < 0.001). Using OLS on logged dollars, with only the presence of thyroid disorders as the independent variable, we found that among individuals with diabetes, presence of thyroid disorders was associated with 64.4% greater total expenditures compared to those without thyroid disorders.

With respect to types of expenditures, similar patterns were observed for outpatient, prescription drug, and other expenditures with presence of thyroid disorders among individuals with diabetes. However, the percent increase associated with thyroid disorders varied from as high as 170.7% for other expenditures to as low as 18.6% for emergency room expenditures.

Table 4 summarizes the parameter estimates, standard errors and *P* values of parameter estimates from separate OLS regressions on logged values of total and types of expenditures among individuals with diabetes with and without thyroid disorders. We also present percent change in expenditures associated with presence of thyroid disorders (last column of Table 4). In the first model, in addition to presence/absence of thyroid disorders, we also included demographic (sex, age, and race/ethnicity). After controlling for these variables, the association between presence of between thyroid disorders and total healthcare expenditures remained significant. However, presence of thyroid disorders was associated with 43.8% greater total expenditures compared to those without thyroid disorders. When additional demographic factors (marital status, area of residence), economic factors (education, family poverty status), access to care factors (health insurance), lifestyle risk factors (smoking, physical activity), and health status factors (perceived mental and physical health) were controlled in Model 2, those with thyroid disorders had 34.3% greater total expenditures compared to those without thyroid disorders. 

When cooccurring chronic conditions were entered into the model, those with thyroid disorders had 23.7% greater total expenditures compared to those without thyroid disorders. In the final model, when macrovascular comorbid conditions were included, the association between presence of thyroid disorders and total expenditures remained highly significant (beta = 0.19 which translated to 21.4% change in expenditures, *P* < .001).

When examined by type of expenditures, however, for emergency-room-related expenditures, the presence of thyroid disorders was non-significant with the addition of chronic cooccurring conditions in Model 3. In the addition, the presence of thyroid disorders was inversely associated with emergency room expenditures with addition of macrovascular comorbid conditions in Model 4.

## 4. Discussion

The current paper estimated incremental healthcare expenditures associated with thyroid disorders among individuals with diabetes. Individuals with diabetes and thyroid disorders had greater healthcare expenditures compared to individuals with diabetes without thyroid disorders, even after adjusting for demographic, economic, lifestyle risk factors, health status, number of cooccurring chronic conditions, and macrovascular comorbid conditions. Our estimates can be considered as an underestimate of the true economic burden because we did not include productivity lost due to missed work days and disability days due to thyroid disorders. Our exploratory analysis suggested that employed individuals with diabetes and thyroid disorders had greater number of work-loss days compared to employed individuals without thyroid disorders and diabetes. These findings indicate that the economic burden among individuals with thyroid disorders may even be greater than suggested by our estimates. 

There are plausible reasons as to why direct healthcare expenditures may be greater for those with thyroid disorders and diabetes compared to individuals with no thyroid disorders and diabetes. For example, different types of thyroid diseases and management of thyroid disorders (i.e., thyroidectomy or radioiodine or medical therapy) may increase the direct healthcare expenditures because these procedures can be very resource-intensive [40]. Future research needs to examine the contribution of specific procedures in thyroid management to the economic burden of individuals with thyroid disorders and diabetes.

A closer examination of factors explaining the incremental direct healthcare expenditures associated with thyroid disorders revealed that nonmodifiable risk factors such as age, gender, and race/ethnicity explained 29.7% the incremental expenditures. After adjustment of other demographic and socioeconomic characteristics, access to care, lifestyle risk factors, perceived physical health, and perceived mental health, the presence of thyroid disorders was associated with nearly one-fifth (18.7%) greater total expenditures. These findings suggest that lifestyle risk factors such as smoking, obesity, and physical activity can be modified to reduce economic burden of thyroid disorders among individuals with diabetes. 

Approximately one-fifth of incremental (21.6%) expenditures were explained by cooccurring chronic conditions. This is not surprising given that three-quarters of individuals with diabetes and thyroid disorders reported cooccurring conditions such as arthritis, depression cancer, and others compared to approximately one-half individuals with diabetes without thyroid disorders. Our findings suggest high comorbidity burden among individuals with thyroid disorders and diabetes. In addition, these findings highlight the need for providers to focus on comanagement of thyroid disorders, diabetes, and other cooccurring chronic conditions to reduce the economic burden of these individuals.

In terms of types of expenditures, it is interesting to note that there was no significant association between thyroid disorders and emergency room expenditures when controlled for chronic cooccurring condition. When macrovascular comorbid conditions were additionally entered into the model, the association was negative. Individuals with thyroid disorders had 2% lower expenditures compared to those without thyroid disorders. These findings suggest that excess emergency room expenditures due to thyroid disorders may be driven by cooccurring chronic conditions macrovascular comorbid conditions. It is also well documented that emergency room visits are associated with a greater number of chronic conditions and/or macrovascular comorbid conditions [41–43]. 

Our study findings have implications for healthcare management strategies for individuals with diabetes. With the growing economic burden of diabetes, healthcare management needs to focus on reducing excess economic burden associated with thyroid disorders among individuals with diabetes. These steps may include early detection and treatment of thyroid disorders. However, there is no systematic approach to screening for thyroid disorders among individuals with type 2 diabetes. Although ADA suggests for screening for thyroid disease at the time of diagnosis of type 2 diabetes [44], screening practices vary widely and pragmatic guidelines are lacking [45]. Our findings highlight the need for developing guidelines in terms of frequency and target population such as women and the elderly for thyroid screening among individuals with diabetes [44]. Furthermore, future studies need to evaluate cost effectiveness of routine thyroid screening among individuals with diabetes. 

 Our study had many strengths and some limitation. The strengths of our study include nationally representative study of adults with diabetes, large sample size, comprehensive list of variables that can influence expenditures such as cooccurring chronic conditions, health status, and capturing health care expenditures from a variety of sources. There are some limitations also. In MEPS, data on medical conditions and utilization are self-reported and may not be accurate. However, validation of chronic conditions against provider reports suggested that households tended to be accurate for conditions that affect daily life (e.g., diabetes, sensitivity 92.1%; thyroid disorders, sensitivity 85%) [46]. It has been reported that MEPS household respondents accurately reported inpatient hospitalizations but underreport emergency room and outpatient visits. However, such underreporting did not affect behavioral analyses [47]. Severity and duration information for diabetes, thyroid disorders, surgical procedures for thyroid disorders, and other conditions was not available; these may have further narrowed the incremental expenditures associated with thyroid disorders. However, the current study controlled for perceived health status and perceived mental health status. Although steps were taken by the MEPS designers to minimize recall bias (diaries and extensive probes to enhance recall), many variables were self-reported and subject to recall bias. In addition, information of microvascular complications may provide insight nto initial economic burden associated with those conditions in individuals with thyroid disorders. Finally, as our study used a cross-sectional design, therefore, we cannot establish causality. 

## 5. Conclusion

In conclusion, our study may be the first study to use a nationally representative survey to report that thyroid disorders were associated with higher healthcare expenditures among individuals with diabetes. Presence of cooccurring chronic conditions was one of the drivers of economic burden in these individuals. Our findings highlight the importance of comanagement of diabetes, thyroid disorders, and other chronic conditions. Future research need to focus on feasibility and cost effectiveness of routine thyroid screening among individuals with diabetes. 

## Figures and Tables

**Table 1:** Description of sample characteristics of individuals with diabetes by the presence of thyroid disorders from the Medical Expenditure Panel Survey, 2007 and 2009.

	Total sample		No thyroid		Thyroid		Sig.
	*N*	wt%	*N*	Row wt%	*N*	Row wt%
All	4,490	100.0%	3,945	87.8%	545	12.1%	
Sex							
Female	2,519	55.0	2,090	79.7	429	20.3	∗∗∗
Male	1,971	45.0	1,855	93.0	116	7.0	
Age							
21–50 years	1,001	20.5	924	89.9	77	10.1	∗∗∗
51–64 years	1,741	38.2	1,548	88.2	193	11.8	
65 years or older	1,748	41.2	1,473	82.2	275	17.8	
Race/ethnicity							
White	2,079	66.1	1,739	83.7	340	16.3	∗∗∗
African American	1,037	14.4	956	91.4	81	8.6	
Latino	1,023	12.7	927	89.4	96	10.6	
Other	351	6.7	323	91.4	28	8.6	
Marital status							
Married	2,550	59.4	2,280	87.5	270	12.5	∗∗∗
Widowed	696	14.9	567	79.0	129	21.0	
Separated/divorced	799	16.9	703	87.5	96	12.5	
Never married	445	8.8	395	86.0	50	14.0	
Metro							
Metro	3,627	80.4	3,192	86.1	435	13.9	
Nonmetro	863	19.6	753	86.1	110	13.9	
Education							
Less than high school	1,465	24.3	1,288	85.4	177	14.6	
High school	1,418	34.2	1,241	85.6	177	14.4	
Above high school	1,565	41.5	1,379	86.9	186	13.1	
Poverty status							
Poor	875	13.9	782	89.1	93	10.9	∗∗
Near poor	1,121	21.5	976	82.8	145	17.2	
Middle income	1,321	30.2	1,145	84.7	176	15.3	
High income	1,173	34.4	1,042	88.1	131	11.9	
Health insurance							
Private	2,346	60.6	2,066	86.8	280	13.2	∗∗
Public	1,706	32.1	1,472	83.4	234	16.6	
Uninsured	438	7.3	407	92.0	31	8.0	
Body mass index categories							
Under/normal weight	721	15.4	647	87.8	74	12.2	
Overweight	1,342	28.4	1,203	87.4	139	12.6	
Obese	2,321	54.2	2,004	84.8	317	15.2	
Missing	106	2.1	91	86.9	15	13.1	
Perceived health							
Excellent/very good	1,033	25.3	922	87.8	111	12.2	
Good	1,674	39.4	1,480	86.2	194	13.8	
Fair/poor	1,783	35.2	1,543	84.7	240	15.3	
Mental health							
Excellent/very good	2,113	49.8	1,867	86.7	246	13.3	
Good	1,607	34.9	1,423	86.0	184	14.0	
Fair/poor	770	15.3	655	84.3	115	15.7	
Current smoker							
Yes	666	14.8	601	89.5	65	10.5	∗∗
Other	3,528	78.9	3,076	85.0	452	15.0	
Missing	296	6.2	268	91.2	28	8.8	
Exercise							
≥3 times a week	1,800	41.1	1,618	87.7	182	12.3	∗
No exercise	2,656	58.9	2,298	85.0	358	15.0	
Missing	296	6.2	268	91.2	28	8.8	

Based on 4,490 individuals with diabetes, aged 21 years or older and were alive during the calendar years 2007 and 2009.

Asterisks represent significant group differences by the presence of thyroid disorders based on chi-square tests.

***P < .001; **.001 ≤ *P* < .01; *.01 ≤ *P* < .05.

Please note that the percentages cannot be calculated by dividing the unweighted number by total sample size because the percentages are derived after accounting for the complex survey design of the MEPS.

**Table 2:** Prevalence of co-occurring chronic conditions by the presence of thyroid disorders among individuals with diabetes from the Medical Expenditure Panel Survey, 2007 and 2009.

	No thyroid disorders		Thyroid disorders		Sig.
	*N*	Col. wt%	*N*	Col. wt%
All	3,945	100.0	545	100.0	
Any macrovascular comorbid conditions	**1,181**	**30.6**	**213**	**39.5**	∗∗∗
Heart disease	1,061	28.0	193	35.8	∗∗
Stroke	225	5.2	41	8.0	∗
Peripheral vascular disease	36	1.0	6	0.8	
Any cooccurring physical chronic conditions	**2233**	**58.5**	**414**	**78.9**	∗∗∗
Arthritis	1635	41.4	313	55.9	∗∗∗
Cancer	398	11.8	91	21.4	∗∗∗
Chronic obstructive pulmonary disease	621	16.3	120	20.5	∗
Gastroesophageal reflux disease	497	13.1	100	17.9	∗
Asthma	380	9.3	77	12.6	∗
Anemia	140	3.1	34	5.9	∗∗
Osteoporosis	103	2.8	33	5.5	∗∗
Endocrine disorders	54	1.6	16	3.0	
Any cooccurring mental health conditions	**929**	**24.9**	**192**	**34.2**	∗∗∗
Depression	623	17.2	137	25.1	∗∗∗
Anxiety	406	10.6	91	15.7	∗∗∗
Schizophrenia	22	0.5	7	0.8	

Based on 4,490 individuals with diabetes, aged 21 years or older and were alive during the calendar years 2007 and 2009.

Asterisks represent significant group differences by the presence of thyroid disorders based on chi-square tests.

***P < .001; **.001 ≤ *P* < .01; *.01 ≤ *P* < .05.

Please note that the percentages cannot be calculated by dividing the unweighted number by total sample size because the percentages are derived after accounting for the complex survey design of the MEPS.

**Table 3:** Average total and type of expenditures (2009$) by the presence and absence of thyroid disorders individuals with diabetes from the Medical Expenditure Panel Survey, 2007 and 2009.

	No thyroid disorders		Thyroid disorders		Unadjusted OLS regression on logged dollars			
	Mean ($)	SE	Mean ($)	SE	Beta	SE	% Change	Sig.
Total	**$11,093 **	**$382 **	**$15,182 **	**$1,055 **	**0.50**	**0.006**	**64.4**	∗∗∗
Inpatient	3,560	250	4,743	808	0.49	0.020	63.1	∗∗∗
Outpatient	3,118	188	4,403	487	0.61	0.012	84.2	∗∗∗
Prescription	3,214	87	4,157	239	0.50	0.005	64.7	∗∗∗
Emergency room	298	26	328	74	0.17	0.006	18.6	∗∗∗
Other	903	52	1552	220	0.99	0.024	170.7	∗∗∗

Based on 4,490 individuals with diabetes, aged 21 years or older and were alive during the calendar years 2007 and 2009.

Asterisks represent significant group differences by the presence of thyroid using ordinary least squares regression log transformed expenditures. Other expenditures included dental, vision, durable medical equipment use, and others.

Percentage expenditures associated with thyroid disorders were estimated by exponentiating the regression coefficients of dummy variables and subtracting one (i.e., percent change = *e* ^*β*^ − 1).

SE: standard error.

***P < .001; **.001 ≤ *P* < .01; *.01 ≤ *P* < .05.

**Table 4:** Intercept and parameter estimates for the presence of thyroid disorders from ordinary least squares regression on logged healthcare expenditures individuals with diabetes from the Medical Expenditure Panel Survey, 2007 and 2009.

Thyroid disease	Beta	SE	% Change	Significance
			(exp(beta)−1)	
Total				
Model 1	0.363	0.005	43.8	∗∗∗
Model 2	0.295	0.004	34.3	∗∗∗
Model 3	0.213	0.005	23.7	∗∗∗
Model 4	0.194	0.006	21.4	∗∗∗
Inpatient				
Model 1	0.389	0.02	47.6	∗∗∗
Model 2	0.299	0.016	34.9	∗∗∗
Model 3	0.2	0.017	22.1	∗∗∗
Model 4	0.152	0.02	16.4	∗∗∗
Emergency room				
Model 1	0.124	0.006	13.2	∗∗∗
Model 2	0.089	0.007	9.3	∗∗∗
Model 3	0.01	0.006	1.0	
Model 4	−0.02	0.005	−2.0	∗∗∗
Outpatient				
Model 1	0.393	0.011	48.1	∗∗∗
Model 2	0.299	0.016	34.9	∗∗∗
Model 3	0.208	0.008	23.1	∗∗∗
Model 4	0.196	0.009	21.7	∗∗∗
Prescription drug				
Model 1	0.348	0.009	41.6	∗∗∗
Model 2	0.282	0.009	32.6	∗∗∗
Model 3	0.193	0.01	21.3	∗∗∗
Model 4	0.179	0.01	19.6	∗∗∗
Other				
Model 1	0.752	0.03	112.1	∗∗∗
Model 2	0.727	0.03	106.9	∗∗∗
Model 3	0.646	0.03	90.8	∗∗∗
Model 4	0.630	0.03	87.8	∗∗∗

Based on 4,490 adults with diabetes, aged 21 years or older and were alive during the calendar year 2007 and 2009. Asterisks represent significant group differences by the presence of thyroid disorders using *t*-tests.

Model 1, included age, gender, and race/ethnicity as independent variables. In Model 2, in addition to the variables used in Model 1, other demographic (marital status and metro status), socioeconomic (education, poverty status, and health insurance) health status (perceived physical and mental health) and lifestyle factors (body mass index categories and smoking status) were included. We controlled for number of cooccurring chronic conditions as an additional variable in Model 3., Finally, in Model 4, we also additionally entered presence of any macrovascular comorbid conditions.

*P < .001; **.001 ≤ *P* < .01; *.01 ≤ *P* < .05.

**Table 5:** CCS codes used to identify cooccurring and macrovascular comorbid conditions from the Medical Expenditure Panel Survey, 2007 and 2009.

Disease category	Clinical classification code(s)
Diabetes	49, 50
Thyroid disorders	48
Any macrovascular comorbid conditions	
Heart disease	96, 97, 100 through 108
Stroke*	342, 430, 431, 432, 434, 435, 436, 437, 438
Peripheral vascular disease	114, 248
Any cooccurring physical chronic conditions	
Arthritis	201, 202, 203, 204
Cancer	11 through 44
Chronic obstructive pulmonary disease	127
Gastro esophageal reflux disease	138
Asthma	128
Anemia*	280 thru 285
Osteoporosis*	206
Endocrine disorders	51
Any cooccurring mental health conditions	
Depression	657
Anxiety	651
Schizophrenia	659

*Indicates used on ICD-9, 3-digit codes instead of CCS codes in defining those conditions.

Details of codes and crosswalk of ICD-9 codes and CCS codes are available on html: http://meps.ahrq.gov/mepsweb/data_stats/download_data/pufs/h128/h128_icd9codes.shtml.

## References

[1] Prevalence and impact of thyroid disease. http://www.thyroid.org/thyroid-events-education-media/about-hypothyroidism/.

[2] Díez JJ, Sánchez P, Iglesias P (2011). Prevalence of thyroid dysfunction in patients with type 2 diabetes. *Experimental and Clinical Endocrinology and Diabetes*.

[3] Perros P, McCrimmon RJ, Shaw G, Frier BM (1995). Frequency of thyroid dysfunction in diabetic patients: value of annual screening. *Diabetic Medicine*.

[4] Papazafiropoulou A, Sotiropoulos A, Kokolaki A, Kardara M, Stamataki P, Pappas S (2010). Prevalence of thyroid dysfunction among greek type 2 diabetic patients attending an outpatient clinic. *Journal of Clinical Medicine and Research*.

[5] Akbar DH, Ahmed MM, Al-Mughales J (2006). Thyroid dysfunction and thyroid autoimmunity in Saudi type 2 diabetics. *Acta Diabetologica*.

[6] Wu P (2000). Thyroid disease and diabetes. *Clinical Diabetes*.

[7] Dimitriadis G, Baker B, Marsh H (1985). Effect of thyroid hormone excess on action, secretion, and metabolism of insulin in humans. *The American Journal of Physiology*.

[8] Dimitriadis G, Mitrou P, Lambadiari V (2006). Insulin action in adipose tissue and muscle in hypothyroidism. *Journal of Clinical Endocrinology and Metabolism*.

[9] O’Meara NM, Blackman JD, Sturis J, Polonsky KS (1993). Alterations in the kinetics of C-peptide and insulin secretion in hyperthyroidism. *Journal of Clinical Endocrinology and Metabolism*.

[10] Okajima F, Ui M (1979). Metabolism of glucose in hyper- and hypo-thyroid rats in vivo. Relation of catecholamine actions to thyroid activity in controlling glucose turnover. *Biochemical Journal*.

[11] Leong KS, Wallymahmed M, Wilding J, MacFarlane I (1999). Clinical presentation of thyroid dysfunction and Addison’s disease in young adults with type 1 diabetes. *Postgraduate Medical Journal*.

[12] Cettour-Rose P, Theander-Carrillo C, Asensio C (2005). Hypothyroidism in rats decreases peripheral glucose utilisation, a defect partially corrected by central leptin infusion. *Diabetologia*.

[13] Maratou E, Hadjidakis DJ, Kollias A (2009). Studies of insulin resistance in patients with clinical and subclinical hypothyroidism. *European Journal of Endocrinology*.

[14] Levin RJ, Smyth DH (1963). The effect of the thyroid gland on intestinal absorption of hexoses. *The Journal of physiology*.

[15] Matty AJ, Seshadri B (1965). Effect of thyroxine on the isolated rat intestine. *Gut*.

[16] Kemp HF, Hundal HS, Taylor PM (1997). Glucose transport correlates with GLUT2 abundance in rat liver during altered thyroid status. *Molecular and Cellular Endocrinology*.

[17] Mokuno T, Uchimura K, Hayashi R (1999). Glucose transporter 2 concentrations in hyper- and hypothyroid rat livers. *Journal of Endocrinology*.

[18] Vaughan M (1967). An in vitro effect of triiodothyronine on rat adipose tissue. *Journal of Clinical Investigation*.

[19] Chen HS, Wu TEJ, Jap TS (2007). Subclinical hypothyroidism is a risk factor for nephropathy and cardiovascular diseases in Type 2 diabetic patients. *Diabetic Medicine*.

[20] Singer MA (2001). Of mice and men and elephants: metabolic rate sets glomerular filtration rate. *American Journal of Kidney Diseases*.

[21] Den Hollander JG, Wulkan RW, Mantel MJ, Berghout A (2005). Correlation between severity of thyroid dysfunction and renal function. *Clinical Endocrinology*.

[22] Yang GR, Yang JK, Zhang L, An YH, Lu JK (2010). Association between subclinical hypothyroidism and proliferative diabetic retinopathy in type 2 diabetic patients: a case-control study. *Tohoku Journal of Experimental Medicine*.

[23] Collet T-H, Gussekloo J, Bauer DC (2012). Subclinical hyperthyroidism and the risk of coronary heart disease and mortality. *Archives of Internal Medicine*.

[24] Bunevičius R, Prange AJ (2010). Thyroid disease and mental disorders: cause and effect or only comorbidity?. *Current Opinion in Psychiatry*.

[25] Dilas LT, Icin T, Paro JN, Bajkin I (2011). Autoimmune thyroid disease and other non-endocrine autoimmune diseases. *Medicinski Pregled*.

[26] Robazzi TC, Adan FF (2012). Autoimmune thyroid disease in patients with rheumatic diseases. *Revista Brasileira de Reumatologia*.

[27] Williams MD, Harris R, Dayan CM, Evans J, Gallacher J, Ben-Shlomo Y (2009). Thyroid function and the natural history of depression: findings from the Caerphilly Prospective Study (CaPS) and a meta-analysis. *Clinical Endocrinology*.

[28] Mitrou P, Raptis SA, Dimitriadis G (2011). Thyroid disease in older people. *Maturitas*.

[29] Gilmer TP, O’Connor PJ, Rush WA (2005). Predictors of health care costs in adults with diabetes. *Diabetes Care*.

[30] Trogdon JG, Hylands T (2008). Nationally representative medical costs of diabetes by time since diagnosis. *Diabetes Care*.

[31] Fu AZ, Qiu Y, Radican L, Wells BJ (2009). Health care and productivity costs associated with diabetic patients with macrovascular comorbid conditions. *Diabetes Care*.

[32] Agency for Healthcare Research and Quality The medical expenditure panel survey: household component full year files, MEPS HC-129 2009 full year consolidated data file. http://meps.ahrq.gov/mepsweb/data_stats/download_data/pufs/h129/h129doc.pdf.

[33] Agency for Healthcare Research and Quality The medical expenditure panel survey: MEPS HC-123: 2009 full year population characteristics. http://meps.ahrq.gov/mepsweb/data_stats/download_data/pufs/h123/h123doc.pdf.

[34] Bureau of labor statistics: consumer price index. http://www.bls.gov/cpi/#tables.

[35] Medical Expenditure Panel Survey use and expenditures related to thyroid disease among women age 18 and older, U.S. noninstitutionalized population. *Statistical Report*.

[36] Rosenzweig JL, Weinger K, Poirier-Solomon L, Rushton M (2002). Use of a disease severity index for evaluation of healthcare costs and management of comorbidities of patients with diabetes mellitus. *American Journal of Managed Care*.

[37] Pearce EN (2011). In people with subclinical hypothyroidism, TSH level >10 mIU/l may predict increased risk of coronary heart disease and related mortality. *Evidence-Based Medicine*.

[38] American Diabetes Association Economic costs of diabetes in the U.S. in 2007. *Diabetes Care*.

[39] Halvorsen R, Palmquist R (1980). The interpretation of dummy variables in semilogarithmic equations. *American Economic Review*.

[40] Zanocco K, Heller M, Elaraj D, Sturgeon C (2012). Is subtotal thyroidectomy a cost-effective treatment for Graves disease? A cost-effectiveness analysis of the medical and surgical treatment options. *Surgery*.

[41] Hospital stays for patients with diabetes, 2008. http://www.hcup-us.ahrq.gov/reports/statbriefs/sb93.pdf.

[42] Woo VC, Carter M, Bialy M, Rehman J Emergency room visits: comparison of individuals with type 1 and type 2 diabetes. http://professional.diabetes.org/Abstracts_Display.aspx?TYP=1&CID=55894.

[43] Egede LE (2004). Patterns and correlates of emergency department use by individuals with diabetes. *Diabetes Care*.

[44] American Diabetes Association (2012). Standards of medical care in diabetes—2012. *Diabetes Care*.

[45] Kadiyala R, Peter R, Okosieme OE (2010). Thyroid dysfunction in patients with diabetes: clinical implications and screening strategies. *International Journal of Clinical Practice*.

[46] MacHlin S, Cohen J, Elixhauser A, Beauregard K, Steiner C (2009). Sensitivity of household reported medical conditions in the medical expenditure panel survey. *Medical Care*.

[47] Zuvekas SH, Olin GL (2009). Validating household reports of health care use in the medical expenditure panel survey. *Health Services Research*.

